# Deep eutectic solvent-based shaking-assisted extraction for determination of bioactive compounds from Norway spruce roots

**DOI:** 10.3389/fchem.2024.1385844

**Published:** 2024-04-02

**Authors:** Alina Kalyniukova, Alica Várfalvyová, Justyna Płotka-Wasylka, Tomasz Majchrzak, Patrycja Makoś-Chełstowska, Ivana Tomášková, Vítězslava Pešková, Filip Pastierovič, Anna Jirošová, Vasil Andruch

**Affiliations:** ^1^ Faculty of Forestry and Wood Sciences, Czech University of Life Sciences Prague, Prague, Czechia; ^2^ Department of Analytical Chemistry, Institute of Chemistry, Faculty of Science, P. J. Šafárik University, Košice, Slovakia; ^3^ Department of Analytical Chemistry, Faculty of Chemistry, Gdańsk University of Technology, Gdańsk, Poland; ^4^ Department of Process Engineering and Chemical Technology, Faculty of Chemistry, Gdańsk University of Technology, Gdańsk, Poland

**Keywords:** deep eutectic solvents, Norway spruce roots, green chemistry, polyphenolic compounds, choline chloride

## Abstract

Polyphenolic compounds play an essential role in plant growth, reproduction, and defense mechanisms against pathogens and environmental stresses. Extracting these compounds is the initial step in assessing phytochemical changes, where the choice of extraction method significantly influences the extracted analytes. However, due to environmental factors, analyzing numerous samples is necessary for statistically significant results, often leading to the use of harmful organic solvents for extraction. Therefore, in this study, a novel DES-based shaking-assisted extraction procedure for the separation of polyphenolic compounds from plant samples followed by LC-ESI-QTOF-MS analysis was developed. The DES was prepared from choline chloride (ChCl) as the hydrogen bond acceptor (HBA) and fructose (Fru) as the hydrogen bond donor (HBD) at various molar ratios with the addition of 30% water to reduce viscosity. Several experimental variables affecting extraction efficiency were studied and optimized using one-variable-at-a-time (OVAT) and confirmed by response surface design (RS). Nearly the same experimental conditions were obtained using both optimization methods and were set as follows: 30 mg of sample, 300 mg of ChCl:Fru 1:2 DES containing 30% w/w of water, 500 rpm shaking speed, 30 min extraction time, 10°C extraction temperature. The results were compared with those obtained using conventional solvents, such as ethanol, methanol and water, whereby the DES-based shaking-assisted extraction method showed a higher efficiency than the classical procedures. The greenness of the developed method was compared with the greenness of existing procedures for the extraction of polyphenolic substances from solid plant samples using the complementary green analytical procedure index (ComplexGAPI) approach, while the results for the developed method were better or comparable to the existing ones. In addition, the practicability of the developed procedure was evaluated by application of the blue applicability grade index (BAGI) metric. The developed procedure was applied to the determination of spruce root samples with satisfactory results and has the potential for use in the analysis of similar plant samples.

## 1 Introduction

Secondary plant metabolites, mainly polyphenolic compounds, play an important role in the processes of growth, reproduction and the defense mechanisms of plants against pathogens and environmental stresses (Hartmann and Trumbore, 2016; Pang et al., 2021). They possess adaptive traits that have undergone natural selection through evolution or climate change. The extraction of secondary plant metabolites is the first step in evaluating phytochemical changes in plants. However, it should be noted that the extracted substances may depend on the extraction method chosen (Bentley et al., 2020). Due to the various environmental factors affecting the synthesis of certain compounds in plants, it is necessary to analyze a large number of samples to obtain statistically significant results, leading to the need to use a large amount of harmful organic solvents for extraction.

In line with the requirements of green chemistry, the current trend is to replace dangerous solvents with less harmful and environmentally friendly alternatives, as well as to reduce the total amount of solvents used. One possibility is the use of deep eutectic solvents (DES), which can be considered as promising green solvents for the extraction of bioactive compounds from complex matrix samples, such as fruits, vegetables and plants (Duan et al., 2016; Shishov et al., 2017; Vieira et al., 2018; Cai et al., 2019; El Kantar et al., 2019; Skarpalezos and Detsi, 2019; Bajkacz et al., 2020; Chen et al., 2020; Ivanović et al., 2020; Koel et al., 2020; Hikmawanti et al., 2021; Kalyniukova et al., 2021; Chen et al., 2022; Mansour et al., 2024). However, the high viscosity of most DESs is a fundamental limitation that can affect the extraction efficiency of the target compounds and subsequently complicate the quantification of analytes by instrumental methods. The addition of water can reduce the viscosity of DESs and thus increase the extraction efficiency (El Achkar et al., 2019; Rozas et al., 2021).

One of the most commonly used types of DES involves systems based on choline chloride as a hydrogen bond acceptor (HBA) in combination with various hydrogen bond donors (HBD), such as organic acids, urea or sugars. Recently, articles have been published devoted to DESs prepared from choline chloride and sugars to the study of the influence of the nature of the HBD (sucrose, glucose, fructose, and xylose) on the distribution behavior of selected compounds in a two-phase liquid–liquid system (Liu et al., 2022); to the use in an ultrasound-assisted dispersive liquid–liquid microextraction procedure for the extraction of aflatoxin M_1_ in milk samples prior to its determination by UV–VIS spectrophotometry (Gürsoy et al., 2022); and as potential absorbents for NH_3_ capture (Li et al., 2020). DESs based on choline chloride and d-fructose in different ratios were prepared, and their physical properties, such as density, viscosity, surface tension, refractive index and pH, were investigated at different temperatures (25°C–85°C). The measured physical properties indicated that DESs have the potential to be used for various applications, including extraction processes (Hayyan et al., 2012).

In the extraction of bioactive compounds from solid plant samples, the mass transfer is usually supported by some auxiliary energy, for example, by ultrasound or shaking. Here are a few examples of using choline chloride-fructose DES for plant sample analysis: Razboršek et al. investigated different natural deep eutectic solvents (NADESs) based on choline chloride for the ultrasound-assisted extraction (UAE) of phenolic compounds from dried chokeberries and found that the highest values of total phenolics and total flavonoids were obtained using the choline chloride-fructose system. Additionally, the results were compared with those obtained using conventional methanol extraction, and their high extraction efficiency was demonstrated (Razboršek et al., 2020). Takla et al. presented a comparison of the potential of NADES and nonionic surfactants with conventional solvents (methanol, ethanol and water) for the ultrasound extraction of alkaloids from plant material. High-performance thin-layer chromatography was used for quantification. The highest extraction yields were obtained using a DES consisting of choline chloride:fructose (5:2) with 35% water. The results showed that a NADES and surfactants were much more efficient in extracting alkaloids than previous methods (Takla et al., 2018). NADESs prepared from choline chloride (ChCl) and sucrose, fructose, glucose and xylose were used for ultrasound-assisted extraction of antioxidants from the halophyte *Polygonum maritimum* L. (sea knotgrass) and compared with conventional solvents, such as ethanol and acetone. The obtained extracts were evaluated for *in vitro* antioxidant properties and profiled using liquid chromatography analysis. The results indicate that a NADES containing ChCl and sucrose/fructose can replace conventional solvents in the extraction of antioxidants from sea knotgrass (Rukavina et al., 2021).

Zhang et al. developed a selective shaking-assisted extraction of astaxanthin ester and free astaxanthin from *Haematococcus pluvialis* by aqueous biphasic systems (ABS) composed of ionic liquids and deep eutectic solutions. ABS composed of tributyloctylphosphine chloride and choline chloride:D-fructose performed the best. The results was compared with organic solvent extraction and prior methods (Zhang et al., 2024). A DES-based pretreatment followed by microwave-assisted hydrodistillation (MAHD) for the extraction of essential oil from dry fruits of white and black peppers was developed. The DES comprised of choline chloride and fructose at a molar ratio of 3:1 was used. The obtained essential oils were analyzed using gas chromatography-mass spectrometry (GC-MS), identifying more compounds than hydrodistillation (Yu et al., 2017). García et al. developed an electrokinetic chromatography method enabling separation of the four stereoisomers of the acetamide herbicide dimethenamid. They tested different anionic cyclodextrins (CDs) based on the use of single and dual CD systems with the addition of ionic liquids and DESs and found that choline chloride-D-fructose, when added to the CDs dual system, enabled separating the four stereoisomers of dimethenamid (Angeles Garcia et al., 2022).

The aim of this work was to develop a novel shaking-assisted extraction method for phenolic compounds from plant samples using a green extraction solvent, a DES based on choline chloride as the HBA and fructose as the HBD. Despite the popularity of ChCl-based DES for analytical purposes, the mixtures of ChCl with Fru are less investigated, especially for plant analysis. That is why we focused on this combination. The parameters influencing the extraction efficiency were investigated by the one variable-at-a-time method (OVAT) and a response surface design (RS) based on face-centered central composite design. The method was subsequently applied to the analysis of Norway spruce root samples in conjunction with LC-ESI-QTOF-MS quantification of polyphenolic compounds.

## 2 Methods

### 2.1 Chemicals

The DES was composed of choline chloride (ChCl) (purity ≥99%) obtained from Gentham Life Science, UK, and D (−)fructose (Fru) (purity ≥99%) purchased from Acros Organics, United States. The solvents, such as methanol (purity ≥99.9%), acetonitrile (purity ≥99.9%) and water (LC-MS grade), were obtained from Honeywell, Germany. The chemical standards were obtained as follows: piceatannol (purity ≥98%), epicatechin (purity ≥98%), procyanidin B1 (purity ≥98%), (−)-epigallocatechin (purity ≥98%) and taxifolin (purity ≥98%) from Chem Faces, China; isorhapontin (purity ≥98%) from Toronto Research Chemicals, Canada; 4-coumaric acid (purity ≥98%) and (+)-catechin (purity ≥98%) from Extrasynthese, France. Ultra-pure water was obtained using a Millipore Milli-Q Plus water treatment system (Millipore Bedford Corp., Bedford, MA).

### 2.2 LC-ESI-QTOF-MS/MS analysis

LC-ESI-QTOF-MS/MS analysis was carried out using an Agilent 1,290 Infinity II system coupled to an Agilent 6546 LC/MS QTOF mass spectrometer (Agilent, United States). The LC separation was conducted using a Zorbax Eclipse Plus C18 column (2.1 × 50 mm, 1.8 µm), (Agilent, United States). The optimal conditions were as follows: mobile phase A consisted of water containing 0.05% formic acid, and mobile phase B consisted of acetonitrile; flow rate, 1.1 mL min^–1^; injection volume, 1 μL; and column temperature, 35°C. For separation of polyphenolic compounds, the gradient elution was set as follows: 0–0.1 min, 95% A; 0.one to eight min, 72% A; 8–9.1 min, 25% A; 9.1–11 min, 95% A.

The determination of polyphenolic compounds (Table 1) was carried out by LC-MS/MS in negative ionization mode. The QTOF parameters were optimized using the standards and were set as follows: scan range 100–1,000 m/z; drying gas temperature, 350°C; sheath gas flow rate, 12.0 L/min; sheath gas temperature, 400°C; capillary voltage, 5.0 kV; nozzle voltage 0.9 kV; fragmentor, 140 V; collision energy at 10, 20 and 40 eV. MS/MS data were acquired at a scan range of 50–800 m/z; retention time window, 0.5 min; isolation window, 1.3 amu and an acquisition rate of two spectra s^–1^. During the analysis, two reference masses (112.9855 and 966.0007 m/z) were continuously measured to mass correction. The data collection was carried out using the Agilent Mass Hunter Acquisition software, and data analysis was performed using Mass Hunter Qualitative Analysis 10.0 and Q-TOF Quantitative analysis (Agilent, United States).

**TABLE 1:** List of compounds monitored by LC-ESI-QTOF-MS/MS.

Compound	Formula	Retention time, min	Theoretical mass	Extracted mass	m/z	Fragments	Mass error, ppm
Taxifolin	C_15_H_12_O_7_	2.96	304.0583	304.0596	303.0499	285.0404	−0.13
						177.0195	
						125.0245	
Catechin	C_15_H_14_O_6_	1.21	290.0789	290.0783	289.0715	245.0819	0.44
						203.0716	
						151.0402	
						125.0244	
						109.0296	
Procyanidin B1	C_30_H_26_O_12_	1.03	578.1419	578.1429	577.1350	451.1019	0.86
						425.0876	
						289.0713	
						161.0243	
						125.0242	
Epigallocatechin	C_15_H_14_O_7_	1.06	306.0740	306.0742	305.0671	261.0788	0.78
						219.0651	
						167.0350	
						125.0242	
4-Coumaric acid	C_9_H_8_O_3_	1.95	164.0474	164.0474	163.0401	119.0504	0.08
						91.0550	
Epicatechin	C_15_H_14_O_6_	1.93	290.0790	290.0794	289.0721	245.0824	1.30
						174.9566	
						125.0243	
Piceatannol	C_14_H_12_O_4_	3.41	244.0738	244.0736	243.0664	201.0554	−0.01
						130.9660	
Isorhapontin	C_21_H_24_O_9_	3.54	420.1417	420.1420	419.1288	257.0819	1.01
						241.0521	
						178.9524	

### 2.3 Sample collection

Norway spruce root samples (*Picea abies* L.) were collected from a depth of 15 cm approximately 1 m from the stem base in the Ore Mts (GPS coordinates 50.65 lat and 13.63 long). The roots were rinsed from the soil, inserted into conical tubes and placed into liquid nitrogen immediately. Samples were freeze-dried, homogenized, passed through 50 µm analytical sieve, and stored at −80°C before LC-ESI-QTOF-MS analysis.

### 2.4 Preparation of the deep eutectic solvents

Choline chloride (HBA) was mixed with fructose (HBD) in different molar ratios (1:1, 1:2, 2:1 and 5:2) with the addition of 30% water w/w to reduce the viscosity, and the mixtures were stirred at 350 rpm at 60°C on a heating magnetic stirrer (Witeg MSH-20D, Germany) until a transparent liquid was formed.

### 2.5 General extraction procedure

A 30 mg sample was precisely weighed in 2-mL test tube and 300 mg of DES was added. Then, 200 µL of water was added, and the sample was vortexed and placed into the thermoshaker (Bioer, China) for 30 min at 500 rpm. After extraction, the sample was centrifuged at 13,000 rpm for 5 min; 200 µL of collected supernatant was transferred to a new dry test tube, diluted with 800 µL of methanol, filtered through a 0.22 µm PTFE filter and injected into the LC system.

### 2.6 Response surface design

In order to validate findings from OVAT optimization, the response surface design was performed. The selected face-centered central composite design with five replicates on the center point was used to find optimal values of shaking speed, extraction time and extraction temperature. The outcome of the RS was a maximizing of the area under the peak of procyanidin B1, catechin, epicatechin and taxifolin. The RS was performed using Minitab v. 17.1.0 (LEAD Technologies, Inc.), and surface plots were generated using in-house Python code. The residues distribution of the obtained models were checked to determine their close to normal distribution characteristics. The residues distribution for catechin and procyanidin B1 were found to be asymmetric; thus Box-Cox transformation of the model input data for those compounds was proposed (the ‘find optimal λ′ option was implemented) giving satisfactory results. The RS is presented in detail in the Supplementary Materials.

### 2.7 Green assessment tools

The complementary green analytical procedure index (ComplexGAPI) (Płotka-Wasylka and Wojnowski, 2021) was used as the green assessment tool. In addition to the green character evaluation, an assessment of the method practicality was performed. For this purpose, the blue applicability grade index (BAGI) (Manousi et al., 2023) was applied.

## 3 Results and discussion

Parameters affecting the extraction efficiency, such as the choline chloride and fructose molar ratio, shaking speed, extraction time, extraction temperature and addition of water, were investigated.

### 3.1 Effect of choline chloride and fructose molar ratio

**FIGURE 1:**
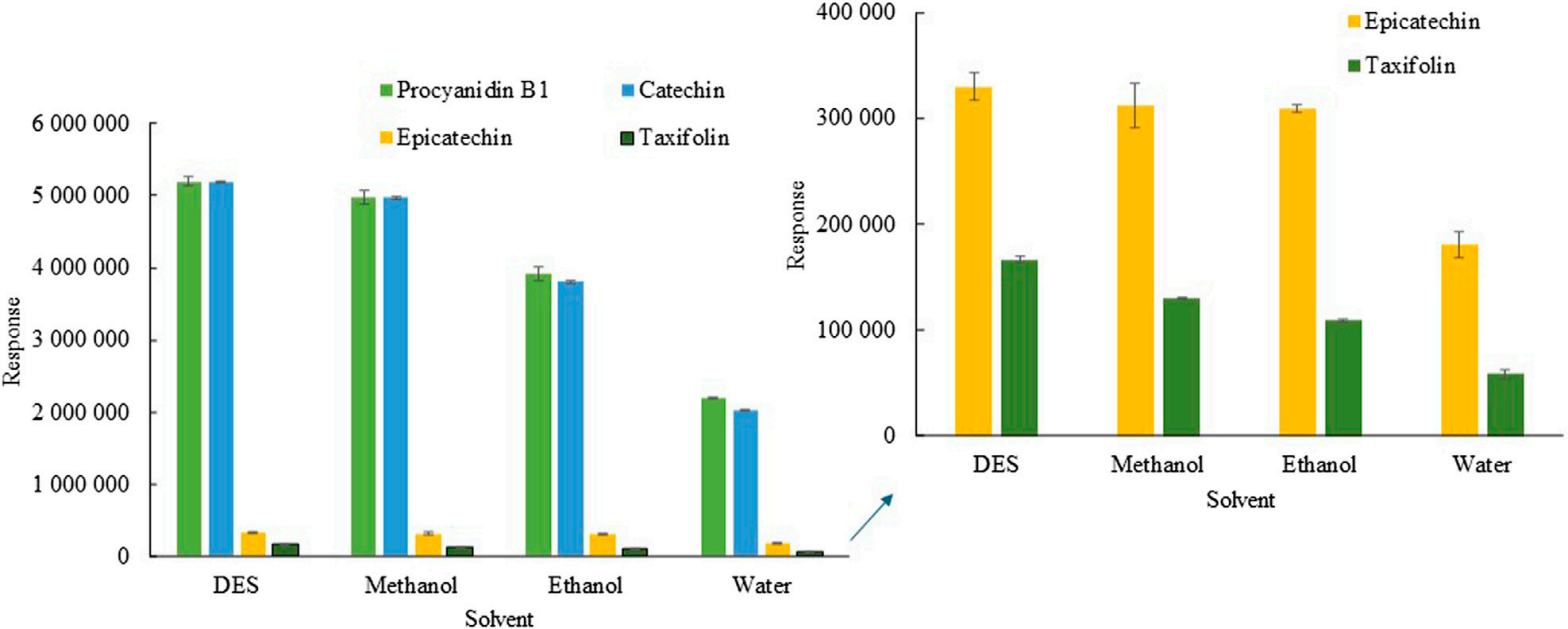
Comparison of DES extraction ability with conventional solvent. Extraction conditions: sample amount, 30 mg; DES amount, 300 mg; solvent amount, 500 μL, water volume, 200 µL (in the case of DES); extraction temperature, 10°C; shaking speed, 500 rpm, extraction time, 5 min.

In general, we can consider DESs as promising solvents in extraction and separation processes (Zainal-Abidin et al., 2017). However, the ability of a DES to extract bioactive compounds from plant samples depends on various factors, such as the type of HBA and HBD used as well as their molar ratio. Therefore, ChCl:Fru DESs with various molar ratios were tested for the extraction of polyphenolic compounds from spruce roots. Supplementary Figure S1 shows that the highest extraction yield was obtained for the DES at a 1:2 M ratio; therefore, this ratio was selected for further studies. The results indicate that an increase in the fructose content within the DES structure correlates with enhanced extraction efficiency. This can be attributed to the presence of active groups in the fructose structure, notably five -OH groups and one = O group, capable of forming robust hydrogen bonds with bioactive substances. In contrast, the ChCl structure possesses only one hydroxyl group, leading to a reduction in extraction efficiency. Another influential factor affecting extraction efficiency is dynamic viscosity, impacting the mass transfer process. With an elevation in the concentration of ChCl, the viscosity of the DES increases, consequently leading to a decrease in extraction efficiency. The extraction efficiency of DES was compared with conventional solvents, such as methanol, ethanol and water. Figure 1 shows that the extraction efficiency of DES is higher compared to methanol at 4%–22%, at ethanol 15%–25%, at 42%–58%, depending on bioactive compounds.

### 3.2 Effect of shaking speed

Another parameter that influences the extraction yield is shaking speed, which ensures the rate of mass transfer from solid phase into the DES phase. The shaking speed was examined in the range from 100 to 1,500 rpm. From Supplementary Figure S2 is evident that the shaking speed has no significant effect on the extraction efficiency; however, a slight increase was observed at 500 rpm which then remains stable up to 1,500 rpm. Therefore, 500 rpm was selected for further optimization steps.

### 3.3 Effect of extraction time

The effect of extraction time was examined in the range of 5–60 min. Supplementary Figure S3 reveals that the extraction yield slowly but gradually and clearly increases with time, reaching a maximum value at 30 min, then remains stable. This can be attributed to the heightened solubility of bioactive compounds in the DES during agitation, thereby facilitating the efficient mass transfer of bioactive compounds from the spruce roots to the DES phase. To ensure high extraction yield in the shortest possible analysis time, 30 min was chosen as the optimum value.

### 3.4 Effect of extraction temperature

Temperature can affect the extraction efficiency; therefore, its influence was studied in a wide range from 5°C to 90°C. In principle, elevating the extraction temperature is expected to decrease the dynamic viscosity of DES, thereby enhancing the efficiency of the mass exchange process. However, excessively high temperatures are undesirable, because the interactions between the DES and bioactive compounds involve an exothermic reaction, in accordance with Van ‘t Hoff’s law. This law asserts that in an exothermic reaction heat is liberated, resulting in a negative net enthalpy change. This, in turn, directly affects the partition coefficient value between the DES and bioactive compounds (Tellinghuisen, 2006). Furthermore, heightened temperatures can lead to the degradation of D-fructose. This degradation may manifest as caramelization followed by pyrolysis of the sugars (Woo et al., 2011).

As can be seen from Supplementary Figure S4, the efficiency of catechin extraction clearly increases with increasing temperature up to 30°C and subsequently decreases. In the case of procyanidin B1, the extraction efficiency is highest and stable up to 20°C; with a further increase in temperature, a lowering of the inter-sample repeatability is observed (30°C–60°C) and at temperatures above 60°C, the extraction efficiency decreases. The extraction efficiency of epicatechin is highest at lower temperatures up to 20°C, and from 30°C it gradually decreases. The extraction efficiency of taxifolin is less affected by temperature, but even in this case we can observe a gentle and slow increase to a maximum of 30°C, followed by a slow but clear decrease. Therefore, choosing the optimal extraction temperature is difficult, and it was necessary to choose a temperature that would provide the best results for the maximum number of analytes. Therefore 10°C was selected for further analysis.

### 3.5 Effect of water volume

In general, DESs are viscous, which complicates their use for the extraction of substances from solid samples such as plants. The addition of a small amount of water can affect the viscosity, but also the density and polarity of the DES, which can lead to improved extraction properties (El Achkar et al., 2019). On the other hand, an excessive amount of water can weaken the interactions between the DES and the target compounds, as well as the interactions between the DES components, which can lead to disruption of the DES supramolecular structure (El Achkar et al., 2019; Vilková et al., 2020). Therefore, the effect of adding water up to 600 µL was investigated and based on the obtained results (Supplementary Figure S5), 200 µL was selected for further experiments.

### 3.6 Results of experimental design

**FIGURE 2:**
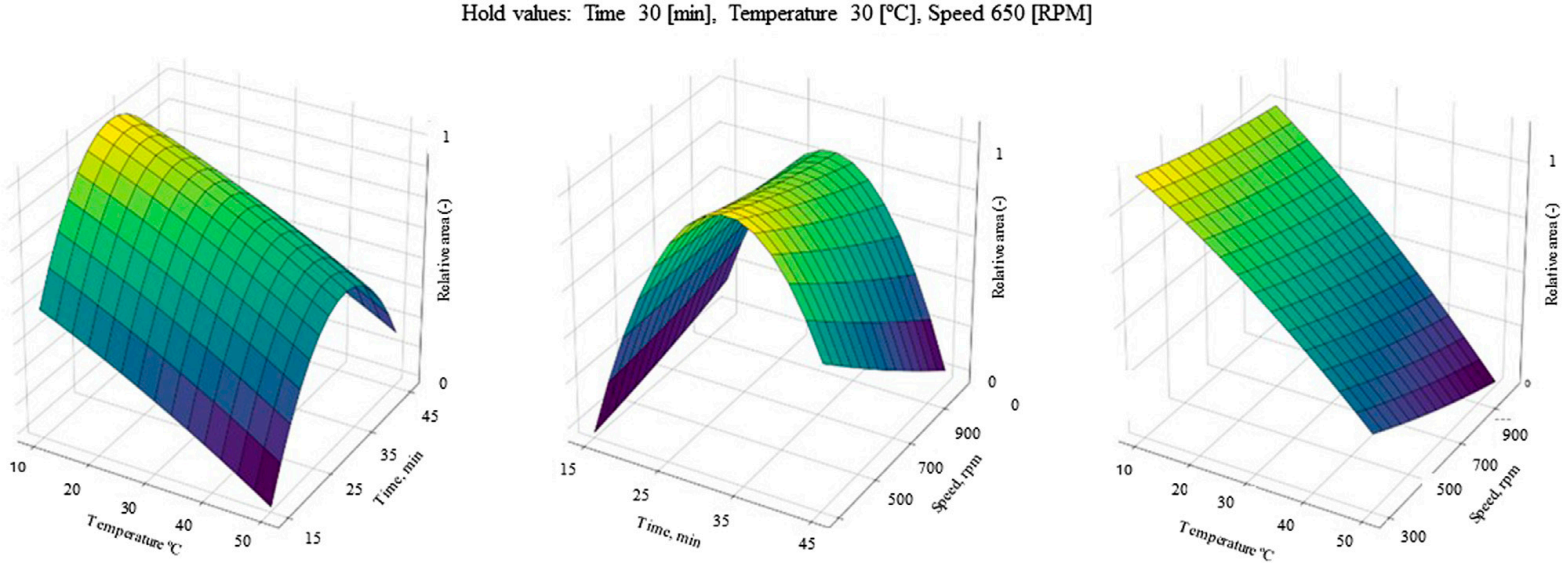
RS surface plots for catechin peak area optimization. Factors taken for optimization: extraction time (15–45 min), extraction temperature (10°C–50°C) and shaking speed (300–1,000 rpm).

In order to confirm the optimal conditions, experimental design was applied. Parameters such as extraction time, extraction temperature and shaking speed were selected for optimization, since the results obtained with OVAT did not include interactions between each optimized factor; thus, it needed to be investigated. In Figure 2 the surface plots of catechin RS optimization are depicted (for others, see Supplementary Materials). Based on the calculations, optimal conditions for catechin determination are a shaking time of 32.5 min, a temperature of 10°C and a shaking speed of 300 rpm. These results are very close to the OVAT optimization. However, for all other optimized compounds strong saddle-shape surface plots were obtained. This can justify findings from OVAT optimization, where the impact of shaking speed and time was insignificant. The insignificance of some factors used for RS also results in a low lack of fit (LoF), where only the RS model for catechin (LoF 0.1 > 0.05) and taxifolin (LoF 0.09 > 0.05) were valid. Therefore, the authors decided to rely on the OVAT results and keep shaking for 30 min at 500 rpm in 10°C.

### 3.7 Application to real samples

To show the applicability of the developed method for the extraction of bioactive compounds, a series of experiments was performed by analyzing Norway root samples spiked with standard solutions at a concentration level of 5 μg g^-1^. The precision and trueness were evaluated by inter-day and intra-day (five consecutive days) measurements. The results are given in Table 2. The recoveries were between 83% and 117%, with RSD less than 9.9% for inter-day and between 93% and 111% with RSD less than 14% for intra-day experiments, respectively.

**TABLE 2:** Inter- and intra-day precision and accuracy of phenolic compounds determination in Norway spruce roots (*n* = 5).

Compound	Added, µg/g	Inter-day			Intra-day		
		Found, µg/g	R, %	RSD, %	Found, µg/g	R, %	RSD, %
Procyanidin B1	0	84.2 ± 0.1		0.2	84.0 ± 2.8		3.3
	5	89.8 ± 1.9	117	2.1	88.7 ± 2.2	94.0	2.5
Epicatechin	0	2.75 ± 0.10		3.6	2.71 ± 0.2		7.3
	5	8.12 ± 0.07	107	0.8	8.27 ± 0.50	111	5.9
Catechin	0	60.8 ± 0.3		0.4	59.8 ± 2.1		3.6
	5	64.9 ± 0.8	82.8	1.2	65.3 ± 2.8	110	4.2
4-Coumaric acid	0	3.23 ± 0.05		1.6	3.22 ± 0.42		13
	5	8.23 ± 0.01	100	0.1	8.46 ± 0.45	105	5.3
Taxifolin	0	0.29 ± 0.03		9.9	0.35 ± 0.05		12
	5	5.28 ± 0.03	99.8	0.5	5.40 ± 0.49	101	9.1
Piceatannol	0	0.19 ± 0.01		7.7	0.21 ± 0.03		14
	5	5.68 ± 0.01	110	0.3	5.23 ± 0.43	100	8.3
Isorhapontin	0	897 ± 3		0.3	895 ± 3		0.1
	5	901 ± 1	80.0	0.1	900 ± 1	100	0.1
Epigallocatechin	0	2.77 ± 0.12		4.5	2.71 ± 0.14		5.3
	5	7.04 ± 0.08	85.1	1.1	7.54 ± 0.44	96.6	5.8

R, recovery; RSD, relative standard deviation.

### 3.8 Assessment of greenness

The ComplexGAPI tool was applied for the evaluation of the green character of the developed procedure. This metric not only allows the evaluation of the analytical protocol in terms of its environmental friendliness but also those processes which precede the analytical procedure itself (Płotka-Wasylka and Wojnowski, 2021; Locatelli et al., 2023). And so, the greenness aspect of the following parameters was evaluated: yield, conditions, reagents and solvents, instrumentation and workup, and purification which may occur before analytical protocol. Considering the generated waste, E-factor is calculated and presented in the middle of the lower part of the pictogram. As these elements can be applied to assess the synthesis of NADES, ComplexGAPI seems be perfect for use in the evaluation of the developed procedure.

**FIGURE 3:**
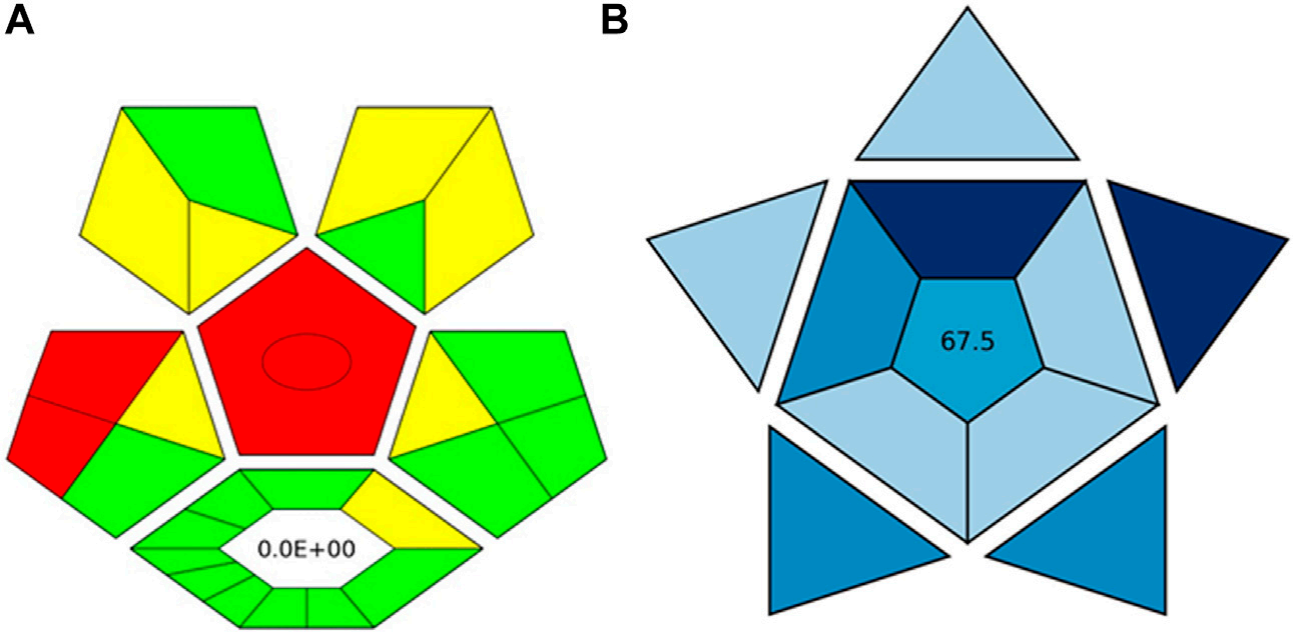
The pictograms of ComplexGAPI **(A)** and BAGI **(B)** generated for the DES-based shaking-assisted extraction coupled to LC-ESI-QTOF-MS for determination of bioactive compounds from Norway spruce roots.

Looking at the generated ComplexGAPI (Figure 3A) pictogram it can be concluded that the DES-based shaking-assisted extraction procedure coupled to LC-ESI-QTOF-MS for determination of bioactive compounds from Norway spruce roots can be considered green. This is mainly because the processes related to the synthesis of the DES are based on non-hazardous reagents. In fact, DES synthesis is a very simple process. The synthesis occurs in a 100% yield and no waste is generated during this process (E-factor = 0). The procedure requires small amounts of reagents for the analytical separation; however, what needs to be noted is that moderate toxic solvents (methanol and acetonitrile) are used here. In addition, a few milliliters of waste per sample are generated. The critical point of the procedure is the necessity to transport and store the sample. In order to show the potential of the developed protocol, the greenness of the developed method was compared with that of existing procedures for the extraction of polyphenolic substances from solid plant samples using the ComplexGAPI approach, and the results for the developed method were better or comparable to existing ones (Table 3).

**TABLE 3:** Comparison of the greenness of the developed method with other reported methods for the extraction of phenolic compounds by means of ComplexGAPI analysis.

Analytes	Sample	Extraction conditions	Detection	ComplexGAPI	Ref.
Catechins	Tea	SLE	UHPLC-UV	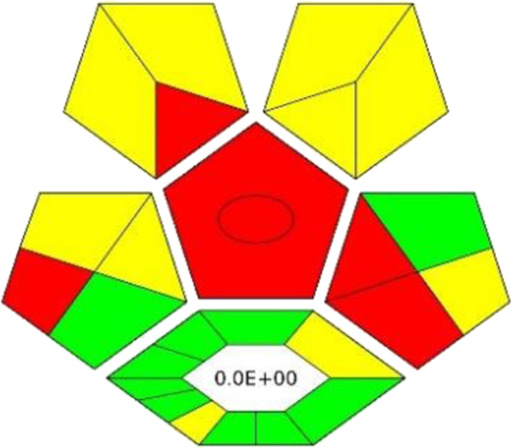	Bajkacz et al. (2020)
		Sample amount: 150 mg			
		DES: Malic acid–Girard’s reagent T 2:1, 30% water; 1.5 mL			
		Time: 50 min			
		Temperature: 50 °C			
Quercetin	Onion	UAE	CSPE	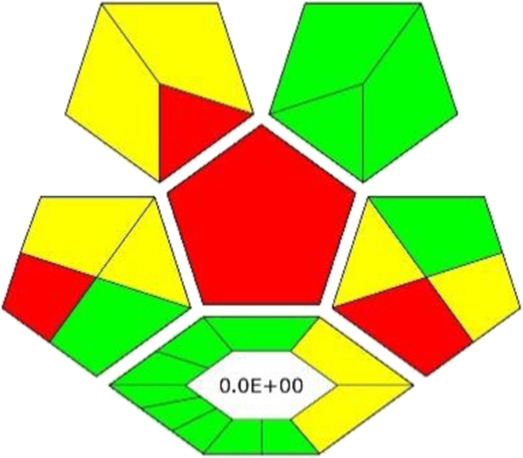	Gomez et al. (2016)
		Sample amount: 50 mg			
		DES: Citric acid–glucose–water 1:1:2; 1 mL			
		Time: 30 min			
Phenolic compounds	Olive leaf	MAE	HPLC-DAD-ESI-TOF-MS	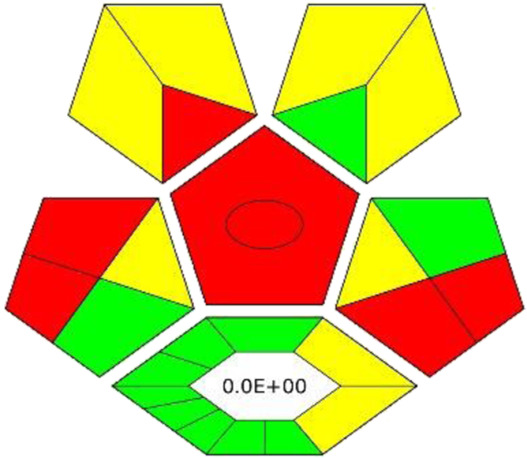	Alañón et al. (2020)
		Sample amount: 200 mg			
		DES: Choline chloride–ethylene glycol 1:2, 43.3% water; 1.5 mL			
		Time: 16.7 min			
		Temperature: 79.6°C			
Quercetin and myricetin	*Ginkgo biloba* leaves	HRE	HPLC-PDA	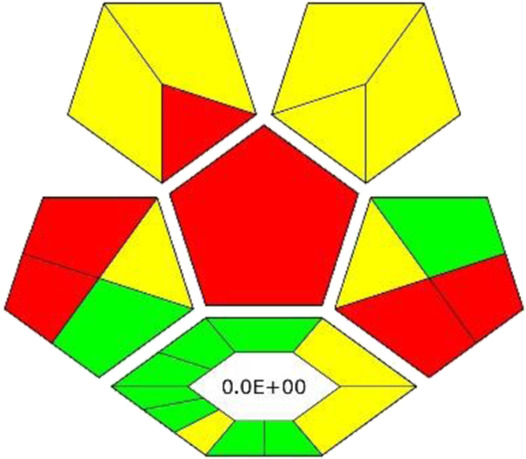	Tang et al. (2017)
		Sample amount: 1 g			
		DES: Choline chloride–oxalic acid–ethylene glycol 1:1:3, 50% water; 10 mL			
		Temperature: 60 °C			
		Time: 30 min			
Quercetin, naringenin, kaempferol, isorhamnetin	*Pollen Typhae*	UAE	HPLC-UV	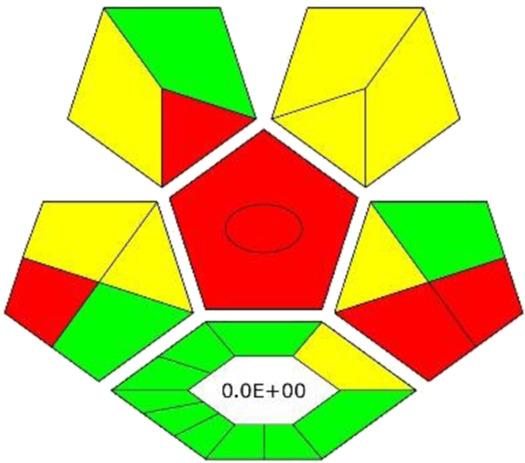	Meng et al. (2018)
		Sample amount: 50 mg			
		DES: Choline chloride–1,2-propanediol 1:4, 30% water; 1 mL			
		Time: 35 min			
Polyphenolic compounds	Norway spruce roots	Shaking-assisted	LC-ESI-QTOF-MS	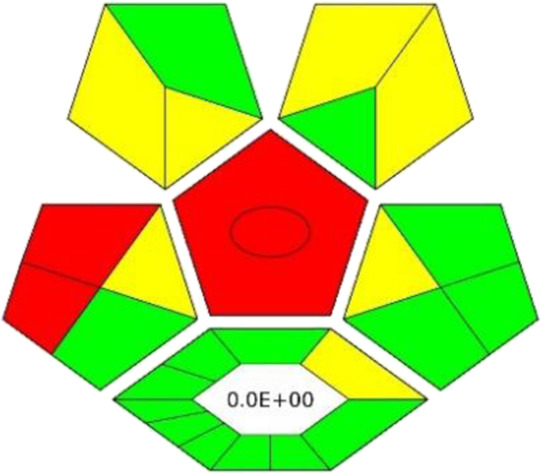	This work
		Sample amount: 30 mg			
		DES: Choline chloride–fructose 1:2, 30% water; 300 g			
		Temperature: 10°C			
		Time: 30 min			

SLE, Solid–liquid extraction; UAE, Ultrasound-assisted extraction; MAE, Microwave-assisted extraction; HRE, heat reflux extraction; CSPE, Carbon screen-printed electrode; UHPLC-UV, ultra-high-performance liquid chromatography with ultraviolet detector; HPLC-DAD-ESI-TOF-MS, High-performance liquid chromatography with photodiode array detector with electrospray quadrupole time-of-flight tandem mass spectrometry; HPLC-PDA, High-performance liquid chromatography with photodiode array detector; HPLC-UV, high-performance liquid chromatography with an ultraviolet detector; LC-ESI-QTOF-MS, liquid chromatography coupled with electrospray ionization quadrupole time-of-flight mass spectrometry.

In addition to the green character evaluation, an assessment of method’s applicability was performed. For this purposes, blue applicability grade index (BAGI) was applied. This metric can be considered complementary to the ComplexGAPI (and other metrics), and it is mainly focused on the practical aspects of White Analytical Chemistry (WAC) (Manousi et al., 2023). Two different types of results can be obtained using the BAGI metric tool and they are correlated to the obtained pictogram and the obtained score as is visible in Figure 3B. In order to be considered practical, it is recommended that the method attains at least 60 points.

Looking at the BAGI pictogram, a great deal of information can be found at first glance. First of all, the information of the analysis was both quantitative and confirmatory due to the employment of the MS detector. The determination enabled the quantification of eight compounds belonging to two different classes (polyphenols and acids). Since the DES needs to be synthesized in the lab in a relatively simple and straightforward way using simple equipment, the procedure lost few points. Regarding the instrumentation, sophisticated equipment was employed. The simultaneous sample preparation can be easily performed using a multichannel pipet. As demonstrated by the results, a one-step preconcentration was needed. The autosampler of LC was used to inject the samples. As for the sample preparation, miniaturized extraction was employed, and the sample volume for the plant matrix was 30 mg. Thus, a BAGI score of 67.5 is attained for the method and the whole protocol shows good applicability potential.

## 4 Conclusion

In conclusion, this study introduced an environmentally friendly shaking-assisted extraction procedure utilizing a deep eutectic solvent based on choline chloride and fructose for the efficient extraction of polyphenolic compounds from Norway root samples coupled with LC-ESI-QTOF-MS detection. For the optimization of extraction efficiency, two optimization approaches were utilized: one-variable-at-a-time and confirmed by response surface design. Almost identical experimental conditions were achieved using both optimization techniques, as follows: 30 mg of the sample, 300 mg of ChCl:Fru 1:2 DES with 30% w/w water content, shaking speed set at 500 rpm, extraction time of 30 min, and an extraction temperature of 10°C. The DES was compared with conventional solvents and the results showed that the extraction efficiency of DES is higher compared to methanol, ethanol, and water. The environmental sustainability of the developed method was evaluated using the ComplexGAPI, while the practicality of the developed procedure was assessed through the blue applicability grade index. Furthermore, the method was successfully applied to the analysis of spruce root samples, yielding satisfactory results. We can assume that the suggested approach can (of course, after appropriate validation) also be used for samples of other plants with a similar structure or for the roots of other plants.

## Data Availability

The raw data supporting the conclusion of this article will be made available by the authors, without undue reservation.

## Abbreviations

ABS, aqueous biphasic systems; CD, cyclodextrin; BAGI, blue applicability grade index; ChCl, choline chloride; ComplexGAPI, complementary green analytical procedure index; DES, deep eutectic solvents; Fru, fructose; HBA, hydrogen bond acceptor; HBD, hydrogen bond donor; NADES, natural deep eutectic solvent; OVAT, one variable-at-a-time method; RS, response surface design; UAE, ultrasound-assisted extraction; MAHD, microwave-assisted hydrodistillation; LoF, lack of fit; WAC, White Analytical Chemistry.
