# Supplementary material for: Deep eutectic solvent-based shaking-assisted extraction for determination of bioactive compounds from Norway spruce roots
Source: Front Chem. 2024 Apr 2;12:1385844. doi: 10.3389/fchem.2024.1385844 (PMC11018933; doi:10.3389/fchem.2024.1385844)
Supplement: Supplementary file 1 [file DataSheet1.docx]

Supplementary Material

Deep eutectic solvent-based shaking-assisted extraction for determination of bioactive compounds from Norway spruce roots

Alina Kalyniukova ^1*^, Alica Várfalvyová ^2^, Justyna Płotka-Wasylka ^3^, Tomasz Majchrzak ^3^, Patrycja Makoś-Chełstowska ^4^, Ivana Tomášková ^1^, Vítězslava Pešková ^1^, Filip Pastierovič ^1^, Anna Jirošová ^1^, Vasil Andruch ^2^

^1^ Faculty of Forestry and Wood Sciences, Czech University of Life Sciences Prague, 165 21 Praha 6 - Suchdol, Czech Republic

^2^ Department of Analytical Chemistry, Institute of Chemistry, Faculty of Science, P. J. Šafárik University, 041 80 Košice, Slovakia

^3^ Department of Analytical Chemistry, Faculty of Chemistry, Gdańsk University of Technology, 80-233 Gdańsk, Poland

^4^ Department of Process Engineering and Chemical Technology, Faculty of Chemistry, Gdańsk University of Technology, 80-233 Gdańsk, Poland

*** Correspondence:
Alina Kalyniukova**
[diuzheva@fld.czu.cz](mailto:diuzheva@fld.czu.cz)


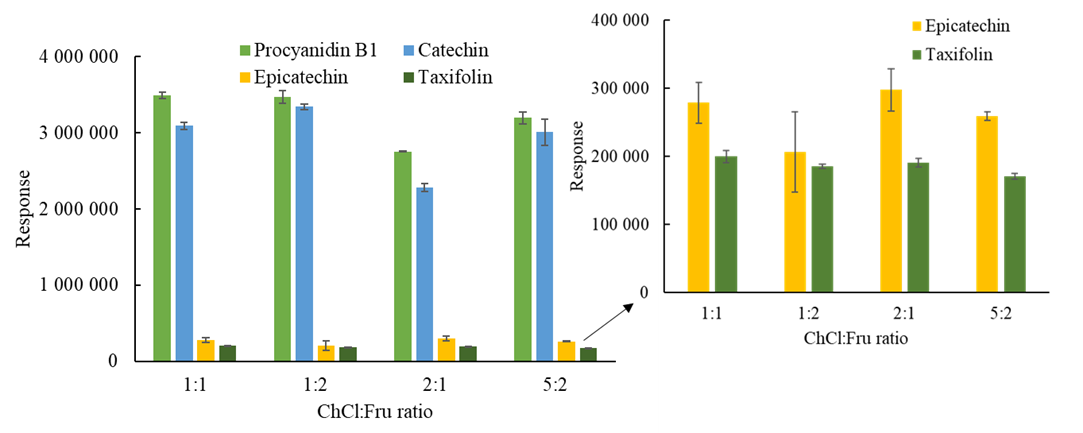


**Fig. S1** Effect of choline chloride:fructose molar ratio. Extraction conditions: sample amount, 30 mg; DES amount, 300 mg; water volume, 200 µL; extraction temperature, 30 °C; shaking speed, 300 rpm; extraction time, 5 min.


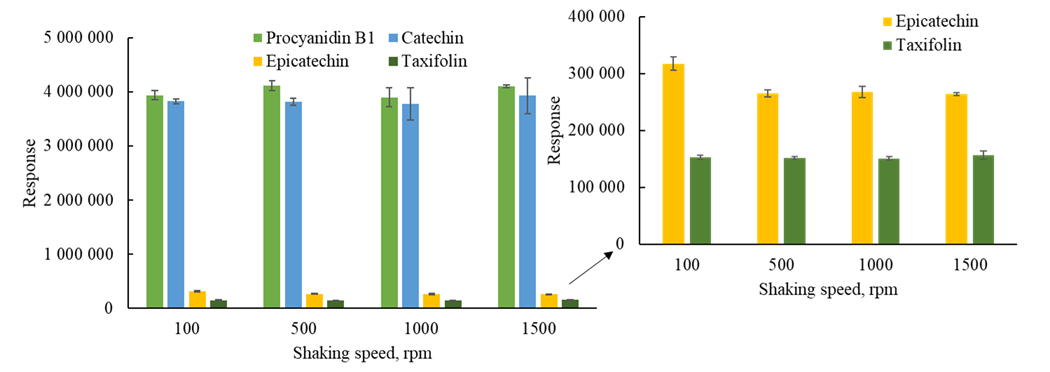


**Fig. S2** Effect of shaking speed. Extraction conditions: sample amount, 30 mg; DES amount, 300 mg; water volume, 200 µL; extraction temperature, 30 °C; extraction time, 5 min.


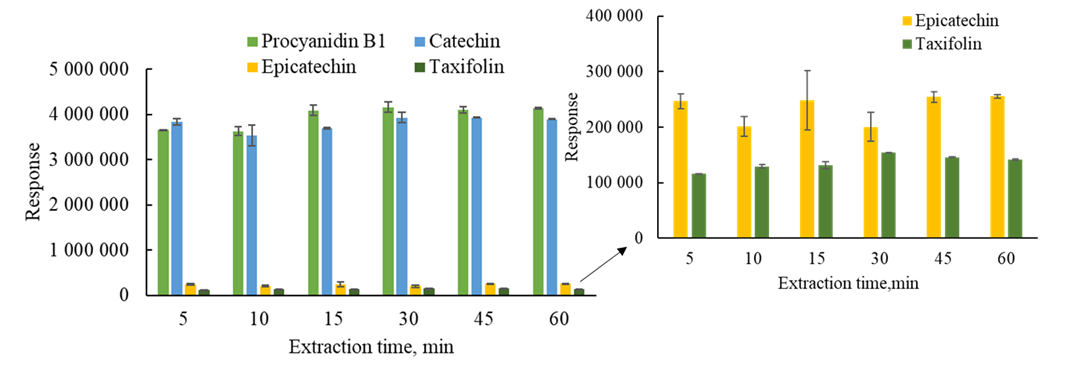


**Fig. S3** Effect of extraction time. Extraction conditions: sample amount, 30 mg; DES amount, 300 mg; water volume, 200 µL; shaking speed, 500 rpm; extraction temperature, 30 °C.


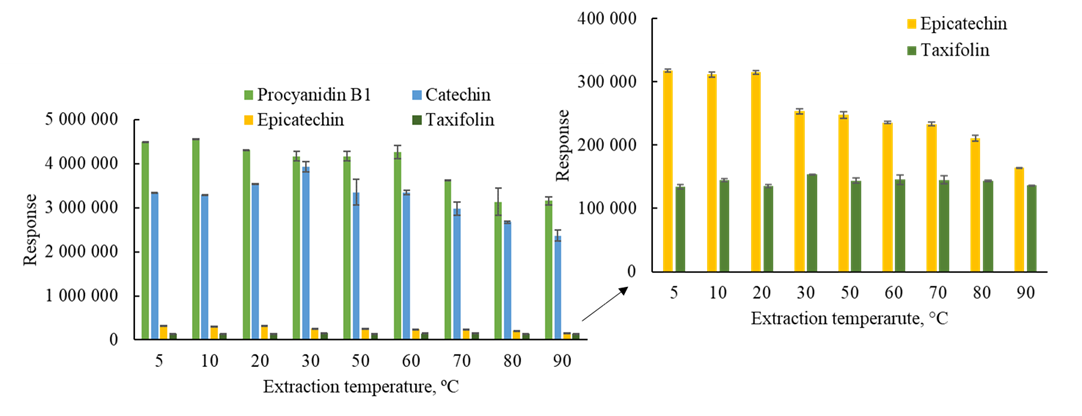


**Fig. S4** Effect of extraction temperature. Extraction conditions: sample amount, 30 mg; DES amount, 300 mg; water volume, 200 µL; shaking speed, 500 rpm; extraction time, 30 min.


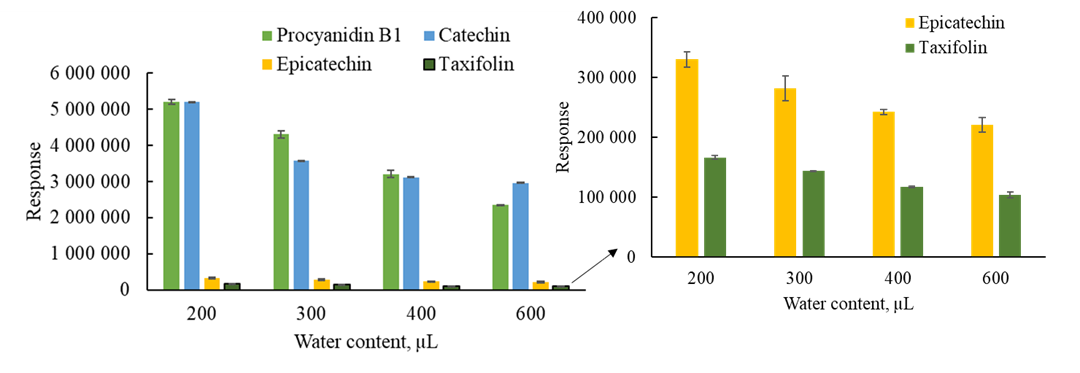


**Fig. S5** Effect of water volume. Extraction conditions: sample amount, 30 mg; DES, amount, 300 mg; shaking speed, 500 rpm; extraction time, 30 min; extraction temperature, 10 °C.

- 1. **RS optimization**

**Table S1**. Optimization matrix.

| Run order | Stand. order | Time [min] | Temp [°C] | Speed [RPM] | Procyanidin B1 [Area×10^6^] | Catechin [Area×10^6^] | Epicatechin [Area×10^5^] | Taxifolin [Area×10^5^] |
| --- | --- | --- | --- | --- | --- | --- | --- | --- |
| 1 | 1 | 15 | 10 | 300 | 4.0 | 3.4 | 2.8 | 1.2 |
| 2 | 3 | 15 | 50 | 300 | 3.9 | 3.1 | 2.4 | 1.5 |
| 3 | 4 | 45 | 50 | 300 | 3.9 | 3.4 | 2.9 | 1.7 |
| 4 | 10 | 45 | 30 | 650 | 4.8 | 3.3 | 2.4 | 1.7 |
| 5 | 15 | 30 | 30 | 650 | 5.9 | 3.5 | 3.2 | 1.6 |
| 6 | 9 | 15 | 30 | 650 | 4.8 | 3.3 | 1.5 | 1.7 |
| 7 | 12 | 30 | 50 | 650 | 3.1 | 3.4 | 2.1 | 1.3 |
| 8 | 13 | 30 | 30 | 300 | 5.6 | 3.4 | 2.4 | 1.3 |
| 9 | 5 | 15 | 10 | 1000 | 4.6 | 3.3 | 3.1 | 1.3 |
| 10 | 17 | 30 | 30 | 650 | 5.8 | 3.5 | 3.2 | 1.6 |
| 11 | 18 | 30 | 30 | 650 | 5.9 | 3.5 | 3.2 | 1.6 |
| 12 | 6 | 45 | 10 | 1000 | 4.5 | 3.3 | 3.2 | 1.5 |
| 13 | 8 | 45 | 50 | 1000 | 3.3 | 3.0 | 1.5 | 1.6 |
| 14 | 2 | 45 | 10 | 300 | 4.6 | 3.4 | 2.9 | 1.3 |
| 15 | 14 | 30 | 30 | 1000 | 5.8 | 3.6 | 3.2 | 1.6 |
| 16 | 19 | 30 | 30 | 650 | 5.9 | 3.4 | 3.7 | 1.6 |
| 17 | 16 | 30 | 30 | 650 | 5.8 | 3.4 | 3.2 | 1.7 |
| 18 | 7 | 15 | 50 | 1000 | 3.1 | 3.1 | 1.6 | 1.6 |
| 19 | 11 | 30 | 10 | 650 | 4.5 | 3.5 | 3.1 | 1.1 |

**2.1. RS surface plots**

***A. Catechin***:

(Catechin [Area]^λ-1)/(λ×g^(λ-1)) = -135573 + 57496 Time [min] - 3316 Temperature [°C]

+ 197 Speed [RPM] - 847 Time [min]·Time [min]

- 26 Temperature [°C]·Temperature [°C] + 0.026 Speed [RPM]·Speed [RPM]

+ 38.3 Time [min]·Temperature [°C] - 8.75 Time [min]·Speed [RPM]

- 1.27 Temperature [°C]·Speed [RPM]

Box-Cox transformation: (λ = 6, g = 3351132 is the geometric mean of Catechin [Area])

Lack of Fit 0.100 (> 0.05)


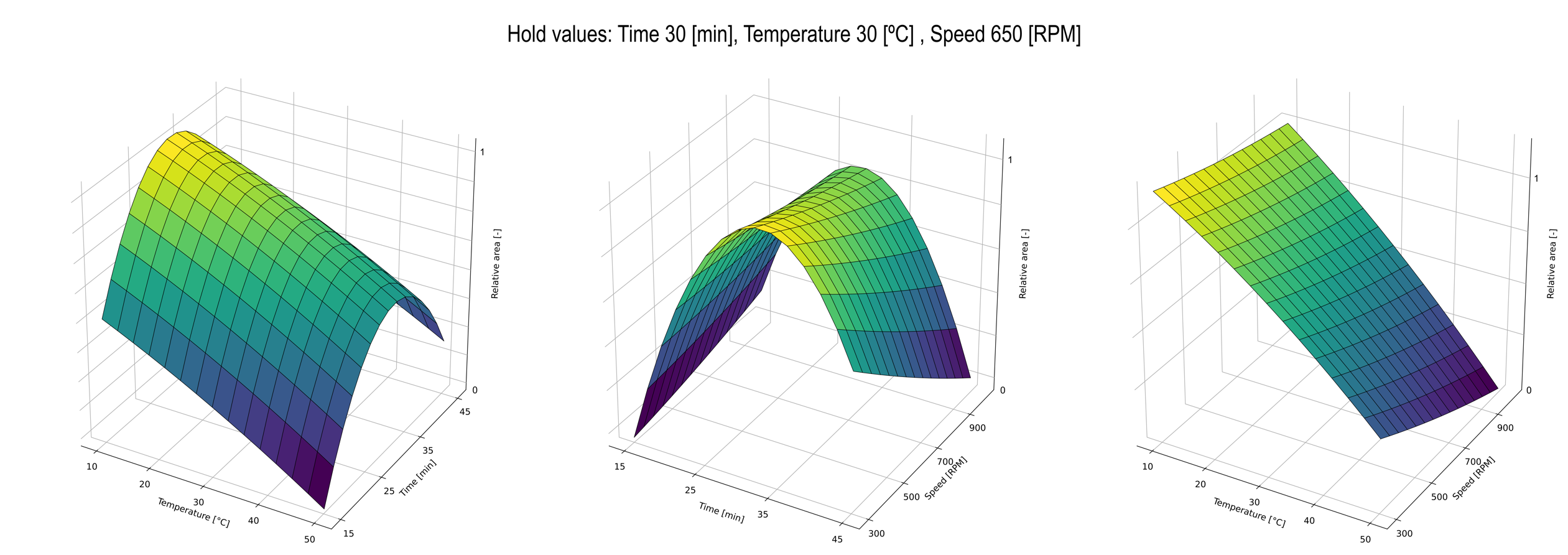


**Figure S6**. RS surface plots for catechin peak area optimization. Shaking parameters taken for optimization: shaking time (15 min - 45 min), shaking temperature (10°C - 50°C), and shaking speed (300 rpm - 1000 rpm).

***B. Epicatechin***:

Epicatechin [Area] = 17247 + 19789 Time [min] + 1352 Temperature [°C] - 19 Speed [RPM]

- 298 Time [min]·Time [min] - 10.9 Temperature [°C]·Temperature [°C]

+ 0.146 Speed [RPM]·Speed [RPM] + 4.5 Time [min]·Temperature [°C]

- 1.69 Time [min]·Speed [RPM] - 4.83 Temperature [°C]·Speed [RPM]

Lack of Fit 0.032 (< 0.05). Model does not fit to the data.


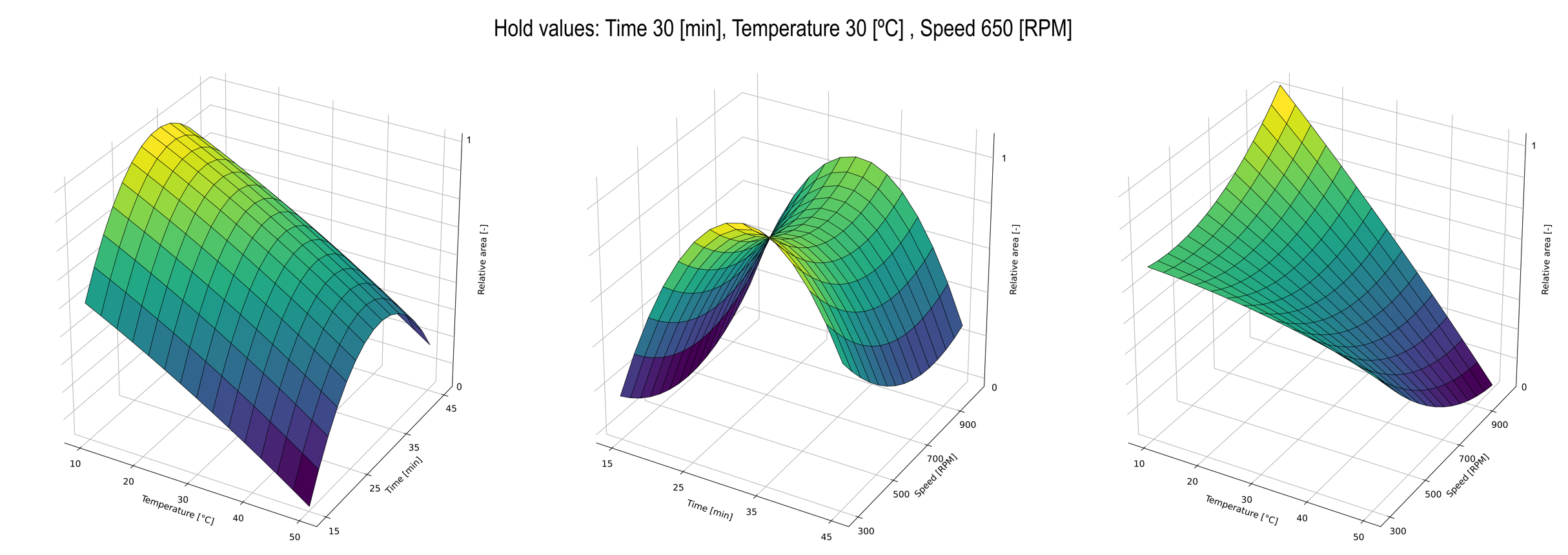


**Figure S7**. RS surface plots for epicatechin peak area optimization. Shaking parameters taken for optimization: shaking time (15 min - 45 min), shaking temperature (10°C - 50°C), and shaking speed (300 rpm - 1000 rpm).

***C. Taxifolin***:

Taxifolin [Area] = 101869 - 4836 Time [min] + 5000 Temperature [°C] + 99.1 Speed [RPM]

+ 88.9 Time [min]·Time [min] - 65.9 Temperature [°C]·Temperature [°C]

- 0.0438 Speed [RPM]·Speed [RPM] + 0.2 Time [min]·Temperature [°C]

- 0.322 Time [min]·Speed [RPM] - 0.610 Temperature [°C]·Speed [RPM]

Lack of Fit 0.090 (> 0.05)


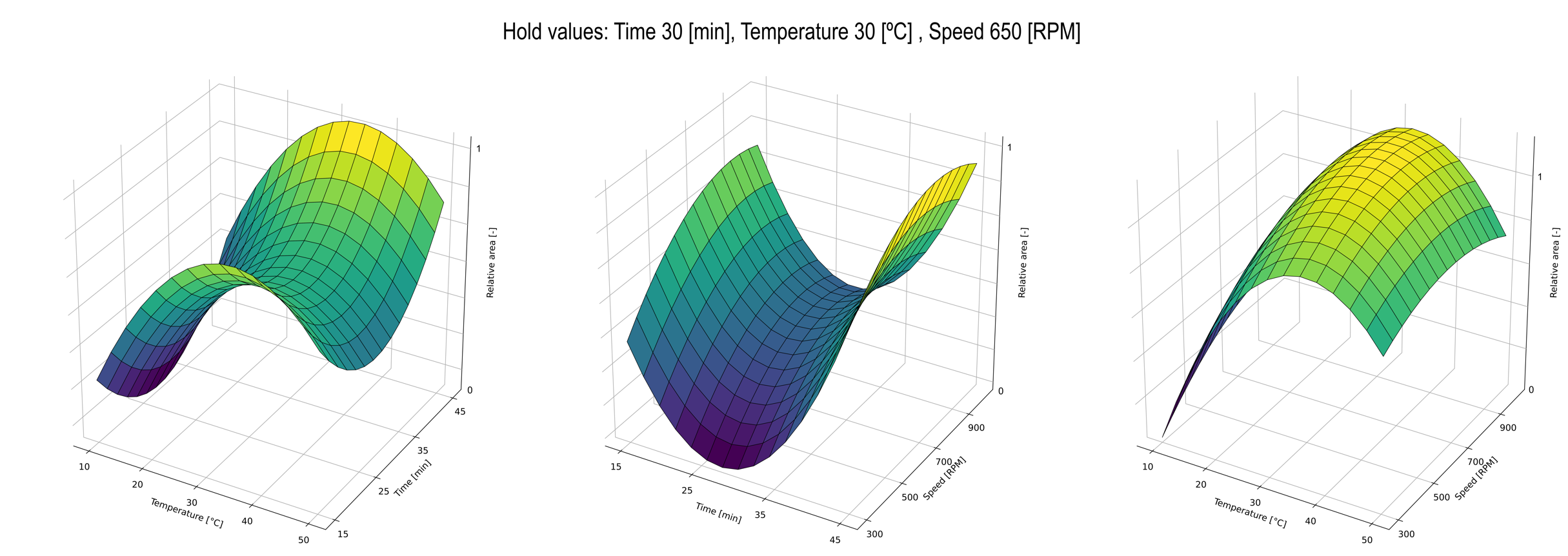


**Figure S8**. RS surface plots for taxifolin peak area optimization. Shaking parameters taken for optimization: shaking time (15 min - 45 min), shaking temperature (10°C - 50°C), and shaking speed (300 rpm - 1000 rpm).

***D. Procyanidin B1***:

Procyanidin B1 [Area]^0.5 = 1342 + 33.7 Time [min] + 54.59 Temperature [°C]

- 0.596 Speed [RPM] - 0.500 Time [min]·Time [min]

- 0.902 Temperature [°C]·Temperature [°C]

+ 0.000653 Speed [RPM]·Speed [RPM]

- 0.0379 Time [min]·Temperature [°C]

- 0.00228 Time [min]·Speed [RPM]

- 0.00868 Temperature [°C]·Speed [RPM]

Lack of Fit 0.000 (< 0.05) Model does not fit to the data.


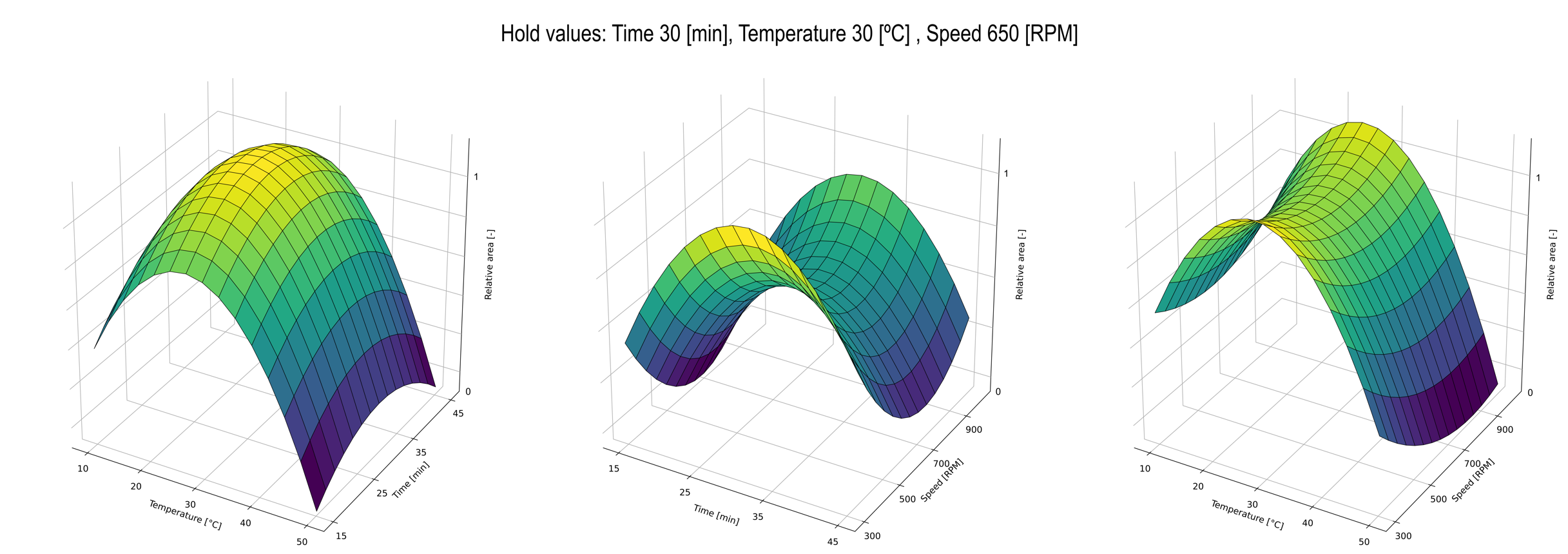


**Figure S9**. RS surface plots for procyanidin B1 peak area optimization. Shaking parameters taken for optimization: shaking time (15 min - 45 min), shaking temperature (10°C - 50°C), and shaking speed (300 rpm - 1000 rpm).
